# Quantitative relationship model between soil profile salinity and soil depth in cotton fields based on data assimilation algorithm: forecasting cotton field yields and profits

**DOI:** 10.3389/fpls.2024.1519200

**Published:** 2024-12-20

**Authors:** Yang Gao, Lin Chang, Mei Zeng, Quanze Hu, Jiaojiao Hui, Qingsong Jiang

**Affiliations:** ^1^ College of Information Engineering, Tarim University, Alar, China; ^2^ Key Laboratory of Tarim Oasis Agriculture (Tarim University), Ministry of Education, Alar, China

**Keywords:** salinization, apparent conductivity, soil conductivity, multivariate linear algorithm, Kalman filter

## Abstract

Soil salinization seriously affects the efficiency of crops in absorbing soil nutrients, and the cotton production in southern Xinjiang accounts for more than 60% of China’s total. Therefore, it is crucial to monitor the dynamic changes in the salinity of the soil profile in cotton fields in southern Xinjiang, understand the status of soil salinization, and implement effective prevention and control measures. The drip-irrigated cotton fields in Alaer Reclamation Area were taken as the research objects. The multivariate linear regression model was used to study the relationship between soil salinity and soil depth in different periods, and the Kalman filter algorithm was used to improve the model accuracy. The results showed that the month with the highest improvement in model accuracy was July, with the model accuracy R^2^ increasing by 0.26 before and after calibration; followed by June and October, with the model accuracy R^2^ increasing by 0.19 and 0.18 respectively; the lowest improvement was in March, which was only 0.01. After the model was calibrated by the Kalman filter algorithm, the fitting accuracy (R^2^) between the predicted value and the actual value was as high as 0.79, and the corresponding RMSE was only 96.17 μS cm^-1^, and the measured value of soil salinity was consistent with the predicted value. Combined with the predicted conductivity data of each soil layer, the total yield of the study area was predicted to be 5,203-5,551 kg hm^-2^, and the income was about 4,953-7,441 RMB hm^-2^. It can be seen that Kalman filtering can improve the prediction accuracy of the model and provide a theoretical basis for studying the mechanism of soil salt migration in drip-irrigated cotton fields at different stages. It is of great significance for evaluating the potential relationship between cotton yield and deep soil salinity and guiding the efficient prevention and control of saline soil in cotton fields.

## 1 Introduction

Cotton is a crucial pillar industry in Xinjiang, particularly in the southern region, with factors such as climatic conditions, seed selection, cultivation management, soil fertility and soil salinity content significantly influencing cotton yields (Zhu et al., 2023). Soil salinity is a primary impediment hindering the growth and development of cotton in this area (Zhou et al., 2021). Excessive soil salinity will accumulate too much sodium and chloride ions, which will antagonize the absorption of essential nutrients such as K^+^, Ca^2+^ and Mg^2+^, leading to nutritional imbalance. Excessive salt accumulation will cause salt stress on the cotton root system, inhibit its use of water, and affect the growth and development of cotton. Under salt stress, cotton leaves will turn yellow or brown, chlorophyll content will decrease, and photosynthesis efficiency will decrease. This directly affects the carbon assimilation capacity of cotton, thereby inhibiting growth and yield. Soil salinization occurs when salts and water in the soil are transported and redistributed under certain natural conditions, such as high temperatures causing evaporation or heavy rainfall, leading to an excessive accumulation of salts in certain areas. Soil moisture is one of the main driving forces of salt migration. Water in the soil drives salt migration through capillary action, osmosis and evaporation. In the linear model, soil salt concentration fluctuates with changes in soil moisture content. El-Naggar et al. (El-Naggar et al., 2021) imaged the conductivity of the soil profile and its relationship with soil moisture patterns and drainage characteristics. Zare et al. (Zare et al., 2020) used EM38 data to perform two-dimensional time-lapse imaging of the soil wetting and drying cycles of flood-irrigated cotton fields. It can be seen that soil moisture has a certain correlation with the conductivity of the soil profile, and soil moisture is an important factor that cannot be ignored in the study of soil salinization.Understanding the salinity content of soil profiles at different depths is critical for studying soil salinization phenomena (Ren et al., 2019; Corwin, 2021; Kalinitchenko et al., 2021). The distribution of salt in the soil profile usually shows obvious depth changes. Especially in the arid areas of southern Xinjiang, when evaporation is strong, the evaporation of surface water drives the migration of salt and accumulates on the surface; and the unique sandy soil has strong permeability, so salt easily migrates to the deep layer with the water flow.Currently, small-area studies can obtain accurate soil salinity profiles through field sampling and indoor analysis (Gits-Muselli et al., 2020; Rahman et al., 2020). However, this method is impractical for medium and large-scale regional studies due to its time-consuming and labor-intensive. Smart agriculture technologies, such as remote sensing satellites, hyperspectral imaging and near-earth sensing, enable the acquisition of spectral information or conductivity values indicative of soil salinity content. By correlating this spectral data with local sampling and laboratory analyses, an inverse model can be constructed to estimate soil salinity in the whole profiles (Schmidt and Bijmolt, 2020; Tancik et al., 2020). This approach significantly improves the efficiency of monitoring soil salinity over larger scales. However, there is a current technology face limitations in achieving high-precision monitoring, particularly for deep soil layers, where remote sensing proves inadequate (Gorji et al., 2019; Wang et al., 2020). Remote sensing technology mainly relies on electromagnetic wave signals, which have limited penetration depth. Optical remote sensing technology, in particular, can only obtain soil information within the range of 0-5 cm soil layer. Even microwave remote sensing, which has slightly stronger penetration ability, generally does not exceed 10 cm. This poses a great challenge to monitoring soil salinity above 20 cm. Thus, there is a pressing need for developing a new approach to accurately estimate and analyze deep soil salinity.

Data assimilation introduced by Charney et al. (Desamsetti et al., 2019) focuses on the distribution of the data and the observation and background field errors, continuously incorporating observations into the model to enhance its accuracy (Geer, 2021). By substituting acquired observations into the model, and the accuracy of predictions is iteratively corrected, resulting in a close correlation with the true value (Ahmed et al., 2020). The primary objective of the data assimilation is to continuously adjust the data model based on the existing data and spatio-temporal distributions, so that the model predicted values align more closely with the actual situation, thus improving prediction accuracy (Zhang et al., 2020; Buizza et al., 2022). Lei et al. (Lei et al., 2020) performed data assimilation of high-resolution thermal and radar remote sensing retrievals for soil moisture monitoring in drip-irrigated vineyards, improving water use assessment and irrigation management. Both surface and root zone soil moisture predicted by the Soil-Vegetation-Atmosphere Transport model were improved by data assimilation, validating its effectiveness for agricultural sub-field scale irrigation management decision. Similarly, Lu et al. (Lu et al., 2021) proposed a data assimilation framework for soil moisture and canopy cover observations to reduce the requirement for parameter calibration in maize simulation using AquaCrop. Their findings indicated data assimilation effectively improved model performance, with joint assimilation outperforming univariate assimilation for accurate maize yield estimation and prediction. Ziliani et al. (Ziliani et al., 2022) incorporated CubeSat data (A small, standardized satellite, typically used for scientific research, technology validation and educational purposes. They can carry a variety of sensors and equipment and collect many types of data) into a crop model to predict crop yields in the early season., which is quite informative for enhancing digital agriculture objectives and improving end-of-season yield prediction. Kalman filter-based algorithms and their variants are extensively used in data assimilation. For instance, Kivi et al. (Kivi et al., 2022) employed the ensemble Kalman filter algorithm to overcome the limitations of process-based models and observational data to improves the accuracy and precision of agricultural forecasts. Huerta et al. (Huerta-Bátiz et al., 2022) used the Soil-Vegetation-Atmosphere Transport (SVAT) model based on an assimilation algorithm with an ensemble Kalman filter to estimate root zone soil moisture throughout a maize growing season in central Mexico. The simulated soil moisture in the top 10 cm of soil is particularly valuable for monitoring the impact of climate change on agricultural production in rain-fed areas, especially in developing countries. Huang et al. (Huang et al., 2017b) monitored soil salinity dynamics at a specific depth (vertical direction) in different soil textures using ensemble Kalman filtering over a 480 m sample zone area, finding that the model performs better in sandy, clayey, and biphasic (sand over clay) soils. Although scholars have achieved good research results, current research on agricultural complex dynamic systems still has certain limitations, and aspects such as multi-time domain, multi-variable situations and regional-scale dynamic prediction have not been explored. For multivariate linear models, the Kalman filter algorithm, as a recursive optimal estimation method, has significant advantages. When model parameters or input data change, Kalman filtering can adjust the estimation results in time without re-adjusting the entire model. It is especially suitable for dealing with multi-variable problems affected by environmental changes, such as prediction of changes in soil salinity over time and moisture.

In this paper, the irrigated cotton fields in the Science and Technology Park of the Twelfth Regiment in Alar City, Xinjiang were used as the study area. The apparent conductivity at different effective depths was used to predict the measured conductivity at different periods and soil depths, and then a multiple linear regression model of measured conductivity and soil depth at each period was established by using the dummy variable processing method, and the model was corrected by the Kalman filtering algorithm to improve the accuracy of the model. In this way, the measured conductivity data can be used to predict the conductivity data at different depths of the soil layer, and then the Kalman filter algorithm can be used to correct the multivariate linear model of conductivity and soil depth. With the help of the above methodology, the relationship between soil depth and profile salinity is investigated, and the soil profile salinity is monitored and predicted in different periods, which provides a reference for planting and quantitative irrigation in cotton fields. This paper focuses on answering the questions of whether the data assimilation algorithm improves the accuracy of the prediction model and how much the accuracy of the prediction model is improved.

## 2 Materials and methods

### 2.1 Study area overview

**Figure 1:**
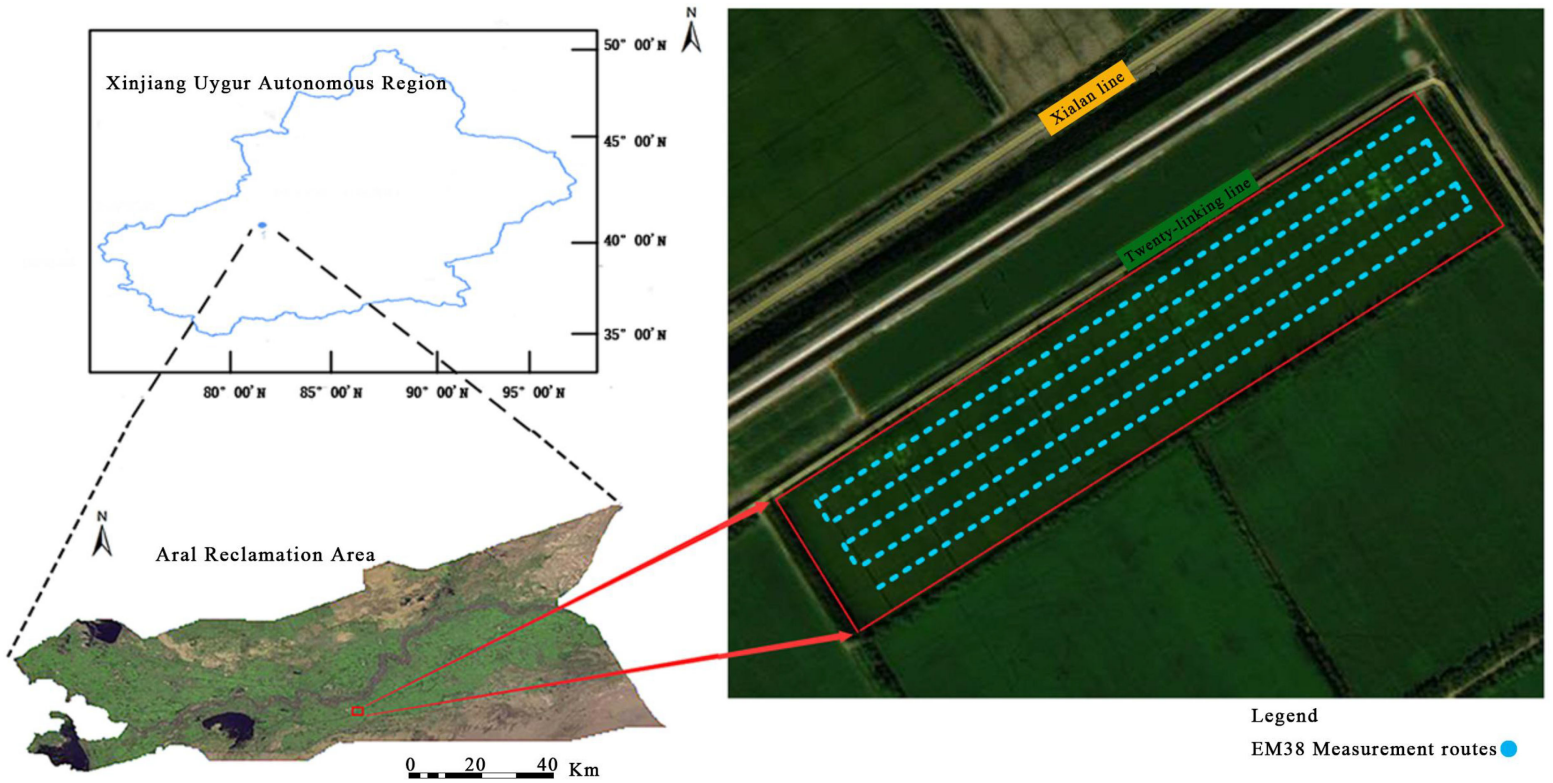
Geographical location map of the study area.

The study area is located within the Twelfth Regiment of Alar Reclamation District, Xinjiang Uygur Autonomous Region. This region (see **Figure 1**) is situated on the south bank of the upper Tarim River, on the northern edge of the Taklamakan Desert, at the center of the Tarim Basin, and directly across the Tarim River from Alar City, the central city of the Corps in southern Xinjiang. The area of the district is 524.8 km^2^, with 318,000 acres of cultivated land, of which 240,000 acres are dedicated to cotton and 78,000 acres to forestry and fruit industry. The elevation of this area ranges from 990 to 1040 m, with the water table fluctuating between 1.4-11.7 m. The primary rainfall period is June through August, the region experiences anthe evapotranspiration ratio of 40:1, characteristic of an extremely arid climate. Rich in light and heat resources, and the frost-free period extends from206 to 220 days. The geographic location of the study area is between 81°29′-81°35′E and 40°48′-40°51′N, covering a total area of 4.45 km^2^. The soil texture type is predominantly sandy loam, and the primary crop cultivated is cotton. A total of 15 hm^2^ of mechanically harvested cotton were selected, located between 40°50′-40°51′N and 80°34′-81°35′E. The dominant cotton variety is Xinluzhong 78, and irrigation water is mainly from the Tarim River and upstream reservoirs. The cotton fields in the study area were drip irrigated five times throughout the growth cycle on 20 June, 8 July, 27 July, 14 August and 28 August, with additional diffuse irrigation on 21 November. The organic matter content is approximately 5.34 g kg^-1^.

### 2.2 Data collection and sampling

**Figure 2:**
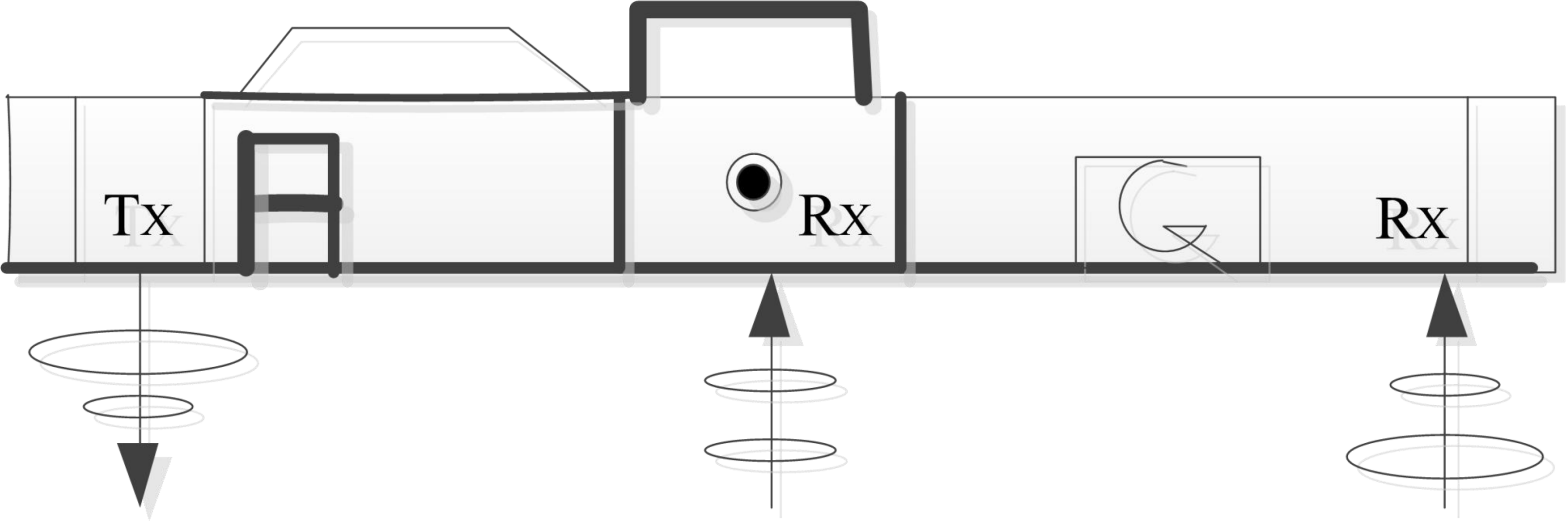
EM38-MK2 simple diagram.

An EM38-MK2 geodesic conductivity meter manufactured by Geonics was used to collect apparent conductivity data (see **Figure 2**) (Kasim et al., 2020). Apparent conductivity and soil samples were collected in March, June, July and October 2021, respectively. The instrument has a transmitting coil (Tx) and two receiving coils (Rx). The distance between Tx and the two Rx is 0.5 m and 1.0 m respectively, so the instrument can measure two kinds of apparent conductivity data under the same model (EC**_x0.5_** and EC**_x1.0_**, X represents mode). The EM38-MK2 instrument can collect apparent conductivity measurement results from both vertical and horizontal directions, generating four different data sets, namely EC**_h0.5_**, EC**_h1.0_**, EC**_v0.5_**, and EC**_v1.0_**. The instrument recording mode record data automatically with the frequency of one point per second from both vertical and horizontal directions. Approximately 1000 sets of apparent conductivity data were collected for each period, with a sampling interval of 1 m. The apparent conductivity data collected in the ‘S’ linear test area were used to determine the best model apparent conductivity data set for different soil layers for linear inverse modeling of soil salinity and its quantitative relationship with soil depth.

Soil samples were systematically collected in compliance with principles of typicality and representativeness, corresponding to the same timeframes as the conductivity data. Employing a stratified random sampling (SRS) approach, samples were collected using soil auger at depths of 0-20, 20-40, 40-60, 60-80 and 80-100 cm. Each sample was promptly placed in a sealed bag, labeled, and transported to the laboratory for soil salinity test.

### 2.3 Soil moisture content and soil electrical conductivity

The soil moisture was determined using the drying method, which involved the following steps: (1) Soil samples were first placed in small aluminum containers and then baked in an oven at 105°C for 2 hours, followed by cooling to room temperature (25°C) in a desiccator. The samples were subsequently weighed, ensuring consistent weightings with an accuracy of 0.001 g). (2) The mixed soil sample was transferred to an aluminum container using an angular spoon, and its mass was recorded using a precision balance. (3) The aluminum container was opened, and the soil was baked in an oven at 105 ± 2°C for 6 h. After removing the lid, the container was cooled in a desiccator to cool to room temperature and immediately weighed.

**Figure 3:**
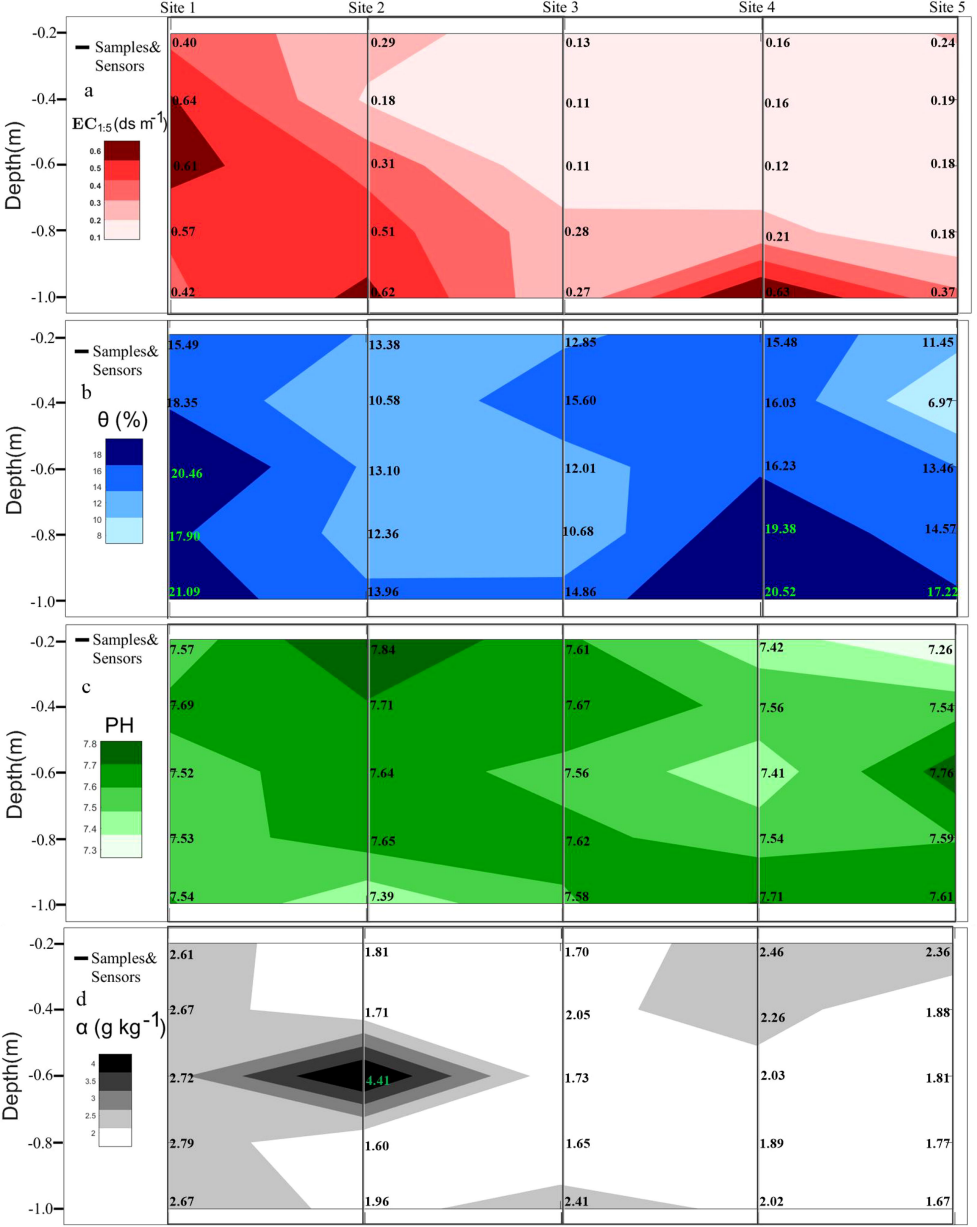
Contour plots of various soil properties measured at five sampling points, including: **(A)** measured electrical conductivity of the soil (EC1:5, dS m^-1^), **(B)** PH value, **(C)** soil water content (θ), and **(D)** salinity content (α g kg^-1^). Data are for reference only and are subject to some uncertainty.

The soil electrical conductivity was measured using the conductivity method (Rooij and Schulz, 2020). The generally high soil conductivity in the study area, such as the 1:1 soil-to-water extracts, often exceeded the measurement range of the assay instrument, rendering direct measurement impractical. Therefore, a water-to-soil mass ratio of 5:1 was typically employed. The mixture was thoroughly shaken, allowed to settle, and the leachate was filtered before determining the electrical conductivity (ECe) using a conductivity meter. To minimize the measurement errors, duplicate samples were prepared for parallel determinations of both moisture content and electrical conductivity. For the five sampling sites in March, measured soil conductivity (ECe), PH, soil sample moisture content (θ) and salinity content (α) were determined, and the nearest-neighbor interpolation method in the MATLAB R2020a Griddata function was utilized to map the depth profile display of the 0.2-1.0 m soil layer at the sampling sites (see **Figure 3**), whereas for the other soil samples only ECe and soil sample moisture content of the soil samples.

### 2.4 Modelling approach

Time series, also known as dynamic series, is a numerical sequence of indicators of a phenomenon arranged in chronological order. Time series analysis can be divided into three major parts, namely, describing the past, analyzing the law and predicting the future (Debella-Gilo and Gjertsen, 2021). Because the time series is a long-term change in the numerical value of an indicator of numerical performance, so the time series of numerical changes behind the inevitable numerical changes in the numerical transformation of the regularity of these regularities is the entry point of time series analysis. In general, the law of change of the value of the time series has the following four kinds of change: (1) long-term trend; (2) seasonal changes in the law; (4) cyclical changes in the law; (4) irregular changes (random perturbation term) (Reddy and Henze, 2011). A time series is often a superposition of the above four types of variability.

The sample series used in this paper are affected by soil depth, and the soil depth adjustment method with dummy variables is utilized to eliminate the effect with soil depth. This soil layer adjustment method is based on the fact that the soil layer depth obeys an additive model Y=T+S+C+I , where Y,T represent aggregate indicators with the same units; and S,C,I produce either positive or negative deviations from the long-term trend. A multiple linear regression prediction model was then developed using soil depth $x_{i}$ and sample *T* as variables:


$$y=\beta_{0}t+\beta_{\text{i}}x_{\text{i}}+C$$

Where, $\beta_{i}$ and *C* for constant coefficient; *y* represents the predicted value of ECe; *t* represents the number of samples; $x_{i}$ represents apparent conductivity.

For sample selection, 12 groups were randomly selected from the 18 groups of measured conductivity data in each period at a ratio of 1:2 as the modeling set, and the remaining 6 groups were used as the validation set. In the above way, time series analysis can help us to understand the change of ECe with soil depth and capture the pattern and trend of observations over time. And it also makes the data smoother and reduces the interference of random fluctuations in the analysis, providing a strong guarantee for the later work.

For the prediction of yield, sample *T* is not considered. The seed cotton yield of the first three years (2016-2018) is used as the modeling set, and the data of the last two years (2019-2020) is used as the validation set. The different soil conductivity data at the bud stage (June) and the boll stage (July) are used as modeling factors to establish a multivariate linear model:


$$y=\beta_{i}x_{i}+C$$

Where, *C* and *β* represents a constant; $x_{1}$ represents 0-20 cm conductivity data.; *x*_2_ represents 20-40 cm conductivity data; *x*_3_ represents 40-60 cm conductivity data; *x*_4_ represents 60-80 cm conductivity data; *x*_5_ represents 80-100 cm conductivity data. In this way, the cotton production of this year can be predicted based on the predicted conductivity data.

### 2.5 Data assimilation

Data assimilation algorithms can generally be classified into two mains: sequential assimilation and continuous data assimilation. The earliest of the sequential data assimilation algorithms is the Kalman filter algorithm, and its idea lays a theoretical foundation for other sequential data assimilation algorithms. The Kalman filter algorithm takes the observed data and the statistical characteristics of the error between the model and the data as the basis, and estimates the state quantities with the restriction of obtaining the minimum error of the state estimation value (Akram et al., 2019). The specific modeling is as follows.

#### 2.5.1 Forecasting


(1) $$X_{k+1}^{f}=M_{k,k+1}X_{k}^{a}$$

where *k* and  k+1 are the two states and the *k*+1 is the next state of k.  f  and *a* represent the predicted and analyzed values, respectively; $X_{k+1}^{f}$ represents the predicted value of *k*+1, which the s$M_{k,k+1}$ represents the linear relationship from *k* state change to *k*+1 state; the $X_{k}^{a}\;$ represents the analyzed value of *k*.


(2) $$P_{k+1}^{f}=M_{k,k+1}P_{k}^{a}M_{k,k+1}^{T}+Q_{k}$$

where $\;P_{k+1}^{f}\;$ and $P_{k}^{a}\;\;$ represent the error covariance matrix between the predicted and analyzed values; $Q_{K}$ is the model error variance matrix. From Equations 1 to 2, it completes the calculation of the pair from *k* state to k+1 state.

#### 2.5.2 Updates

After the observations are acquired in the k+1 state, the corresponding analyzed values and error covariances are updated based on the observations at this moment in time, see Equations 3–5.


(3) $$X_{k+1}^{a}=X_{k+1}^{f}+X_{k+1}\left(Y_{k+1}^{o}-H_{k+1}X_{k+1}^{f}\right)$$

where the superscript *o* represents the observation; $X_{k+1}^{f}$ is calculated from equation 1; $X_{k+1}^{a}\;$ represents the analyzed value at the *k*+1 state; $K_{k+1}$ represents the Kalman gain solved at the *k*+1 state; $Y_{k+1}^{O}$ represents the observation acquired at the *k*+1 state; *H_k_*_+1_ represents the observation operator solved at the *k*+1 state.


(4) $$K_{k+1}={\left(H_{k+1}P_{k+1}\right)}^{T}{\left[H_{k+1}{\left(H_{k+1}P_{k+1}^{f}\right)}^{T}+R_{k+1}\right]}^{-1}$$

(5) $$P_{k+1}^{a}=\left(I-K_{k+1}H_{k+1}\right)P_{k+1}^{f}$$

where $P_{k+1}^{f}$ are calculated from Equation 2, respectively; $P_{k+1}^{a}$ represents the error covariance matrix thatis solved for the analyzed values at the *k*+1 state. Equations 3–5 are based on the observations acquired in the *k*+1 state and adjusted to the background field to obtain the analytical values $X_{k+1}^{a}$ and the error covariance matrix $P_{k+1}^{a}$ at the state and provide the data base for the operations in the next k+2  state.

**Figure 4:**
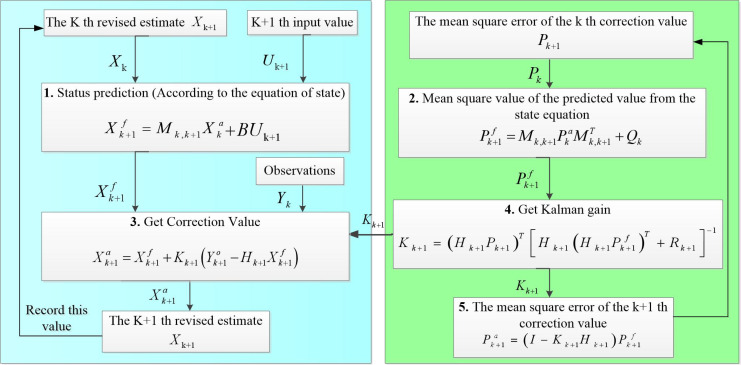
Technical flow chart.

In this paper, the mean value of 0-20 cm soil sample data is set as the initial state; the difference between the first sample value and the mean value is used as the initial error value. The process noise and observation noise are set to white noise, which means that the noise has no dependence on time and is statistically independent of each other. Regarding data preprocessing, soil salinity data generally have significant trends and fluctuate with seasonality, which may cause the data to deviate from Gaussian distribution. In this case, a moving average method is used to remove these components, making the data more stationary and consistent with Gaussian assumptions. For the processing of outliers, use statistical methods to detect and remove outliers to ensure that the data set more closely conforms to the Gaussian distribution. Draw technology flow diagrams (see **Figure 4**).

In contrast to the optimal interpolation algorithm, the Kalman filter algorithm takes into account the state of the model and its changes, and its prediction error varies with the model dynamics process. Compared with the variational algorithm, the Kalman filtering algorithm is easier to implement and its arithmetic process does not need to take into account the accompanying patterns of the model. However, for the use of Kalman filtering algorithms, it is also important to consider whether the data conforms to a Gaussian distribution and whether the state changes conform to linear changes so that the optimal solution can be obtained (Huang et al., 2019). Therefore, whether the Kalman filtering algorithm can be applied in real-world problems requires further model validity tests to be carried out.

### 2.6 Model validation

The quantitative response model of soil depth to profile salinity was developed by firstly establishing a multiple linear regression equation between measured conductivity and soil depth using dummy variable treatment and least squares method, and then calibrating the model using Kalman filter algorithm. After dividing the sample data into modelling and validation sets, they can be validated using cross-validation. Secondly the coefficient of determination R^2^ and the root mean square error RMSE can be used to further judge the effectiveness of the fit between the predicted values and the actual measurements.


(6) $$R^{2}=\sum ({\hat{y}}_{i}-\bar{y}{)}^{2}/\sum (y_{i}-\bar{y}{)}^{2}$$

(7) $$RMSE=\sqrt{\frac{1}{N}\sum {\left({\hat{y}}_{i}-y_{i}\right)}^{2}}$$

Where ${\hat{y}}_{i}$ , *y_i_* and ${\bar{y}}_{i}$ displays the predicted value, true value and average value of the variable, respectively. And the larger the R^2^ value, the smaller the RMSE, the higher the model accuracy, the stronger the predictive ability and the better the stability. On the contrary, the smaller the R^2^ value and the larger the RMSE, the worse the model accuracy and the weaker its predictive ability.

## 3 Results and analysis

### 3.1 Inverse modelling of conductivity in soil layers at different depths

**Figure 5:**
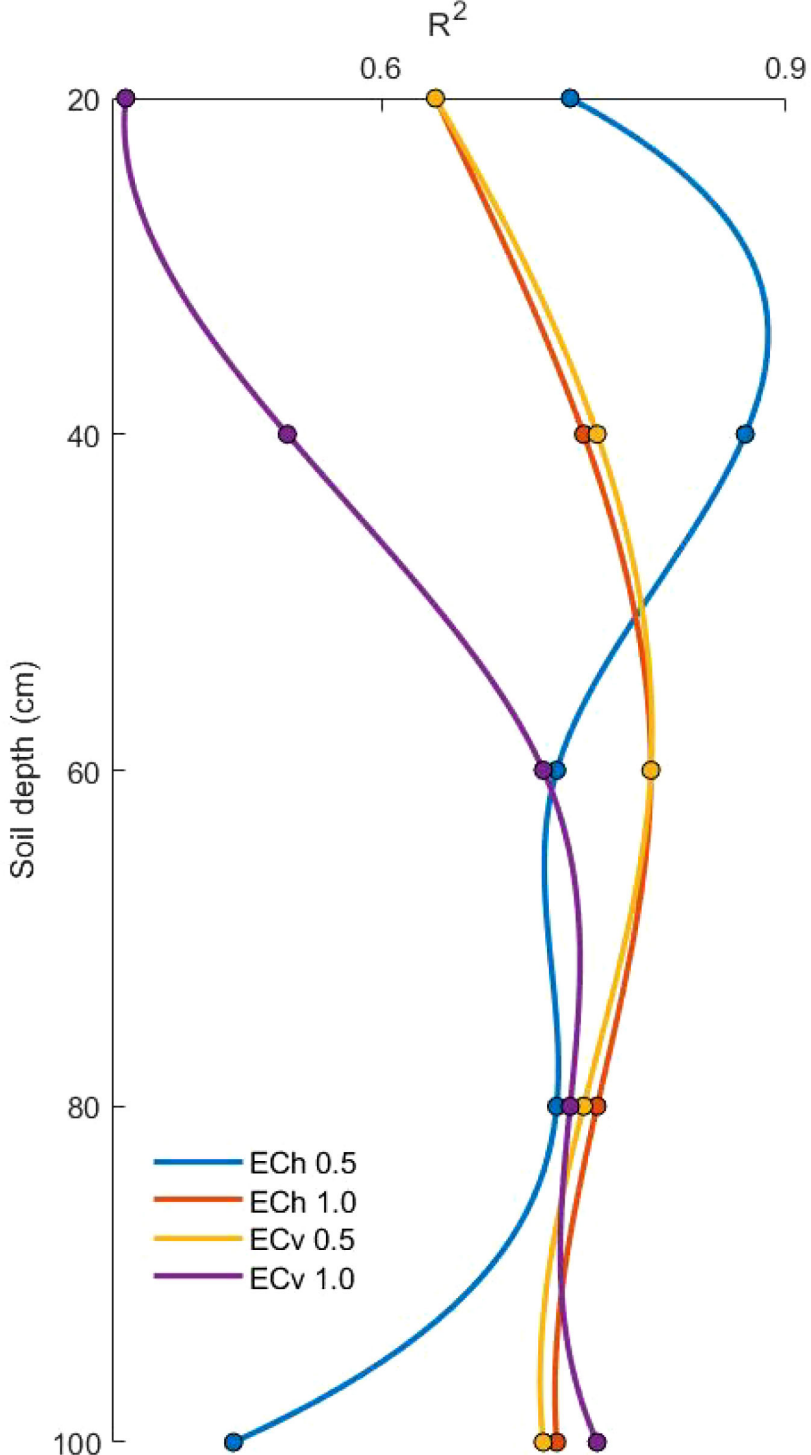
Soil depth determination factor R^2^ for different modelling factors.

The soil conductivity data (ECe) was measured primarily using the saturated paste method on soil samples collected during various periods. Since the detection range of the data collected through the EM38-MK2 is limited to the free state electrolyte content in the deep soil space, the conductivity ECe was used to provide an accurate expression of the salinity status of the agricultural soil, and there was a high correlation between the conductivity EC_e_ collected using the saturated slurry method and the soil salinity. Referring to the research idea of Wang et al., descriptive statistics of soil properties established in the study area for 75 sampling points were carried out considering the sensing range of EM38-MK2 and its degree of response under the change of soil depth under different modes, which can be accessed from the horizontal and vertical aspects of the modelling factors, respectively (Wang et al., 2021). Taking the data collected in June 2021 as an example, the apparent conductivities of 0.5 m and 1.0 m in the horizontal mode and 0.5 m and 1.0 m in the vertical mode were selected as the independent variables, which were denoted by EC_h0.5_, EC_h1.0_, EC_v0.5_ and EC_v1.0_, respectively. With the measured conductivity of different soil depths collected, a linear model was established and the accuracy was compared (see **Figure 5**).

The R^2^ of EC_h0.5_ in the horizontal mode reached a maximum value of 0.87 at 20-40 cm, and thereafter, showed a decreasing trend with increasing soil depth. And the R^2^ of EC_v1.0_ in vertical mode reaches its highest value of 0.76 at 80-100 cm. From the above table, it can be seen that EC_h0.5_ can be chosen as the modelling factor for the 0-40 cm range. Whereas after 40-80 cm, either EC_h1.0_ in the horizontal mode or EC_v0.5_ in the vertical mode can be chosen as the modelling factor. In this paper, EC_v0.5_ in the vertical mode is chosen as the modelling factor. At 80-100 cm, only EC_v1.0_ can be selected as a modelling factor. The results of the analyses in the above table, which are the same as those analyzed by Li et al, are consistent with the response function model between the conductivity data and the change in soil depth in this measurement model (Li et al., 2020).

### 3.2 Effect of soil moisture content on the accuracy of conductivity inversion models

The EM38-MK2 instrument measures the free conductive medium in the soil, and its accuracy is influenced primarily by soil moisture and salinity. To assess the effect of moisture content on model accuracy, soil samples were categorized into three moisture gradients: 0-10%, 10-20%, and >20%. The samples were divided into modeling and prediction sets in a 2:1 ratio, ensuring that the prediction set covered the full range of moisture content. Linear models were constructed to analyze the relationship between apparent conductivity and measured soil conductivity at each gradient.

**Figure 6:**
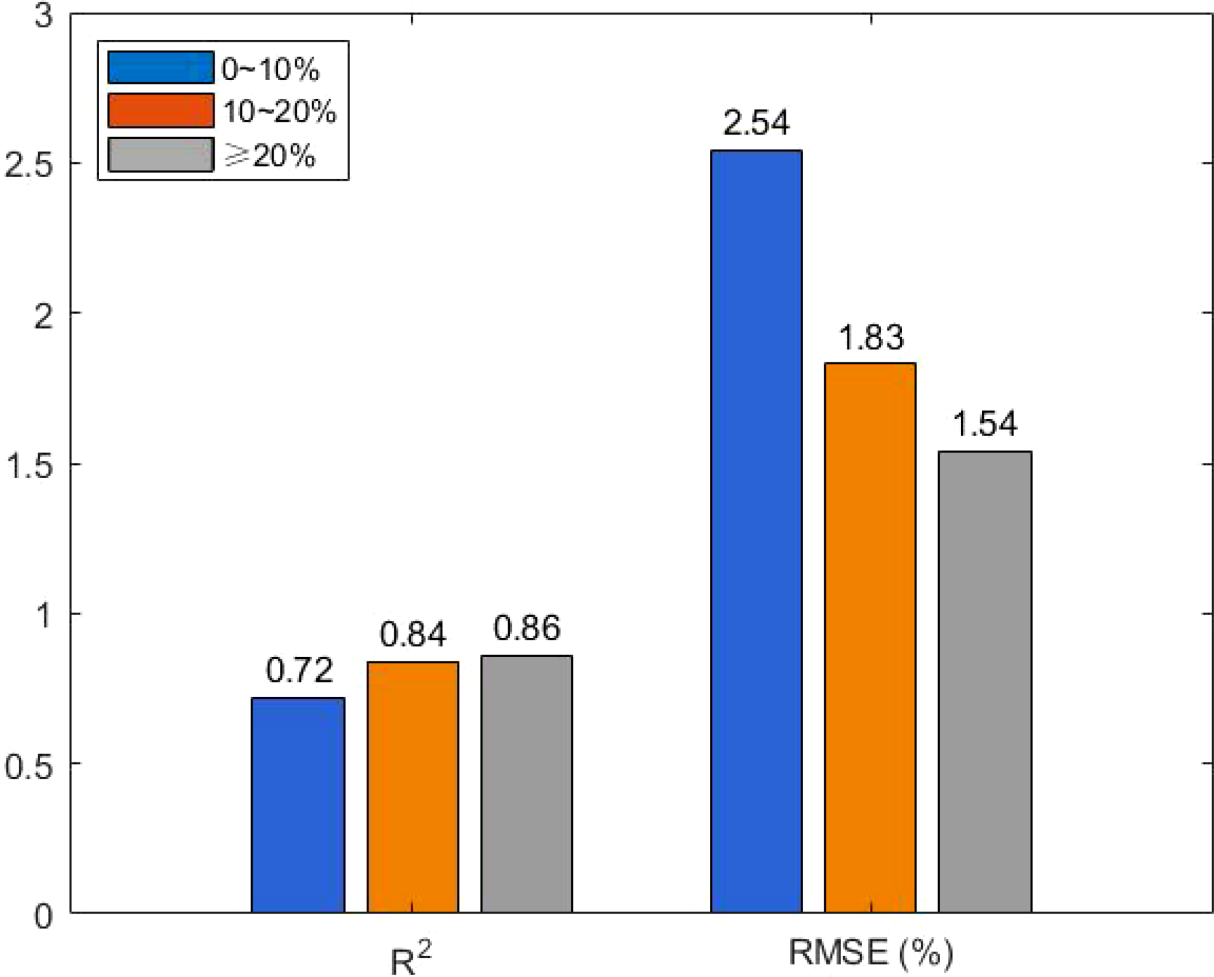
Comparison diagram of model accuracy under different soil moisture gradient.

From **Figure 6**, it can be observed that the model’s R² was 0.72, and the RMSE was largest when soil moisture was in the 0–10% range, indicating poor model accuracy. In contrast, for soil moisture levels between 10–20% and above 20%, the R² values were 0.84 and 0.86, respectively, with smaller RMSE values. These findings suggest that soil moisture levels above 10% have less impact on model accuracy. To ensure model accuracy, soil moisture should be maintained above 10% during modeling. The inverse model was further developed for different periods, and the model accuracy for each period is shown in **Tables 1** and **2**.

**Table 1:** Model of conductivity interpretation in different periods.

	ECe=a+b×EC_h0.5_				ECe=A+B×EC_vx_					
	0-20(cm)		20-40(cm)		40-60(cm)		60-80(cm)		80-100(cm)	
Month	a	b	a	b	A	B	A	B	A	B
March	-0.679	0.907	-0.665	0.834	-0.083	0.503	0.274	0.454	0.325	0.476
June	-0.645	0.769	0.843	0.694	0.264	0.456	0.283	0.463	0.304	0.473
July	-1.566	1.566	-1.424	1.011	-0.675	0.785	0.301	0.496	-0.079	0.521
October	-0.671	0.863	-1.347	1.432	-0.372	0.534	-0.405	0.573	0.353	0.479

a, b, A, B represent constants; ECe represents measured conductivity data; EC_h0.5_ represents 0.5m apparent conductivity data in horizontal mode; EC_vx_ represents 0.5 m and 1.0 m apparent conductivity data in vertical mode.

**Table 2:** Differences in model accuracy for different soil moisture content gradients.

Date	Judging Indicators	0-20(cm)	20-40(cm)	40-60(cm)	60-80(cm)	80-100(cm)
2021/03/10	R^2^	0.81	0.78	0.89	0.86	0.77
	RMSE	0.0673	0.1284	0.0627	0.0671	0.1307
2021/06/03	R^2^	0.71	0.82	0.81	0.80	0.72
	RMSE	2.3875	0.9087	1.9132	0.9284	2.3894
2021/07/07	R^2^	0.79	0.89	0.87	0.88	0.85
	RMSE	0.1387	0.0723	0.0834	0.0915	0.0882
2021/10/19	R^2^	0.77	0.84	0.86	0.85	0.86
	RMSE	0.1289	0.0923	0.0627	0.0804	0.0757

The accuracy of the conductivity interpretation model was compared across different time periods. For the 0–40 cm soil depth range, EC_h0.5_ in the horizontal mode was selected as the modeling factor, while EC_v0.5_ and EC_v1.0_ in the vertical mode were chosen for the 60–100 cm range. The results show that the model’s accuracy and stability were higher in July and October, when soil moisture levels were above 10%, compared to March and June when moisture levels were lower.

### 3.3 Kalman filter algorithm and implementation

In March, June, July and October 2021, a total of four soil sample collections were carried out, each time 18 fixed points were selected to sample the soil profile at different depths, and measured conductivity data were measured for each period. The optimal sampling scheme is selected by comparing the effect of different isotopes on the accuracy of the inversion model, so that the measured conductivity of soil layers at different depths over time can be predicted with the help of the apparent conductivity of soil layers representing different effective depths in different measurement modes. In order to further explore the relationship between soil salinity in the profile and soil depth, the relationship between soil depth and soil salinity in the profile was further investigated with the help of measured conductivity EC_e_ data measured at different depths of the soil layer in each period. Firstly, 12 sets of the 18 sets of measured conductivity data for each period were randomly selected on a 1:2 basis as the modelling set. The sample series used were affected by soil depth, and the effect with soil depth was removed using a soil depth adjustment method with dummy variables, followed by a multiple linear regression prediction model using the least squares method.

From the Kalman filter and its variant forms, it can be seen that the Kalman filter algorithm is more suitable for linear problems; while the extended Kalman filter algorithm can be processed through the Taylor’s formula, which can convert the nonlinear problem into a linear problem to be processed; and the ensemble Kalman filter algorithm is more suitable for the multi-dimensional space which is strongly nonlinear (Khodarahmi and Maihami, 2023). In view of the strong linear correlation between the salt content of the soil profile and the depth of the soil layer at different times and at different depths, the Kalman filter algorithm was chosen to further study the relationship between the salt content of the profile and the depth of the soil layer.

**Figure 7:**
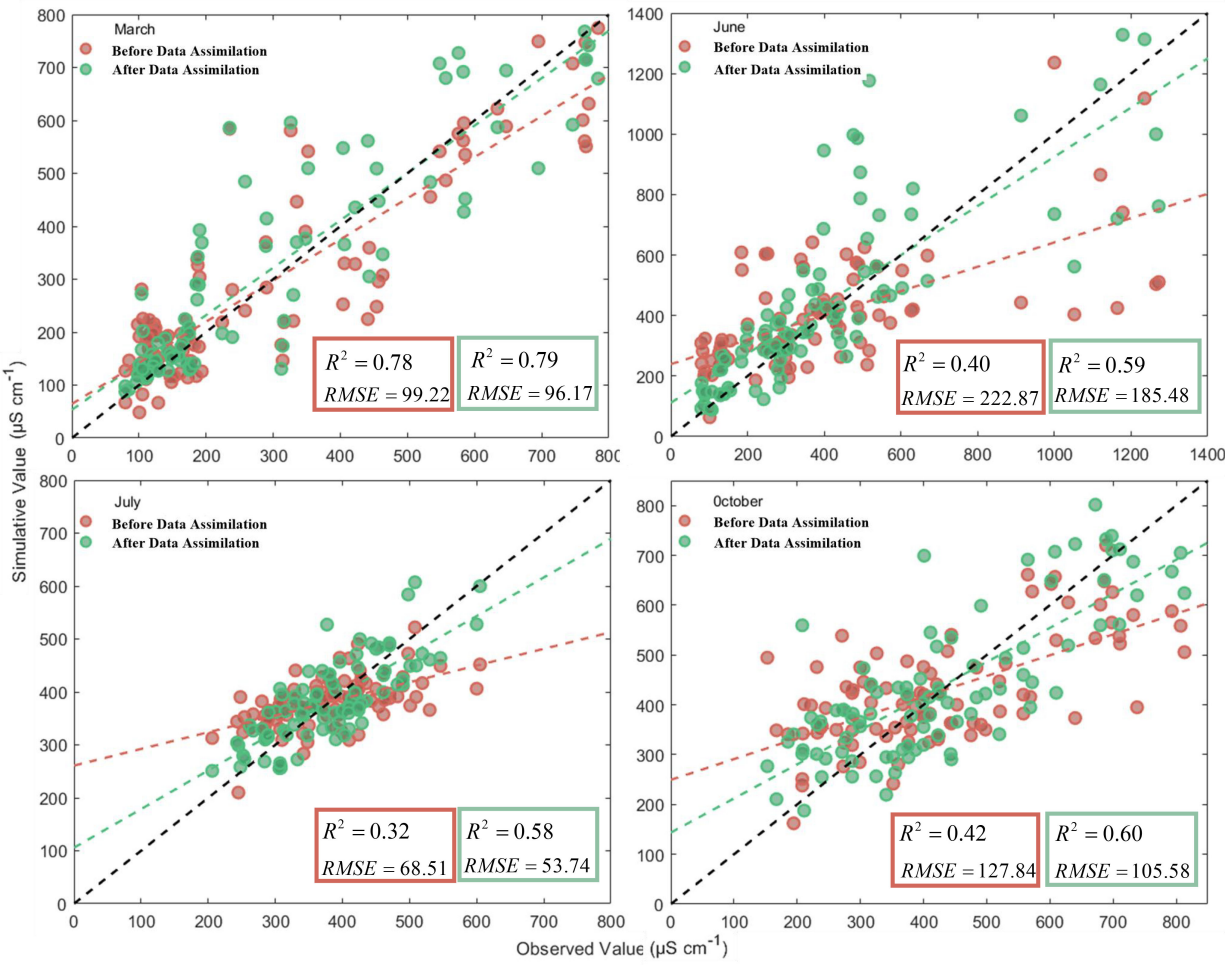
Comparison of observed and simulated values before and after assimilation at different time periods.

Using the Kalman filter algorithm, the multivariate linear inverse prediction models of measured conductivity and soil depth for different periods were calibrated, and the accuracy of the models was all improved to some extent. The best correlation between predicted and measured conductivity data was found in March, with an R^2^ of 0.79. It is significantly higher than the other three months. However, the model accuracy before calibration reached 0.78, with the lowest growth rate. The model-corrected determination coefficients in June and October were slightly higher than that in July, with R^2^ being 0.59 and 0.60 respectively; however, the model-corrected determination coefficient R^2^ in July increased the most, from 0.32 to 0.58, with an increase rate of 81.3%. Therefore, using the dummy variable processing method and the least squares method to establish the multivariate linear inversion model of measured conductivity and soil depth in each period, and then using the Kalman filter algorithm to correct the model, the correlation between the model predicted value and the measured value was obviously improved, and the model prediction effect became better. Comparison of the accuracy of the corrected predicted and measured values for different periods (see **Figure 7**).

In **Figure 7**, it can be seen that the best fit between the corrected best predicted values and the measured values for the month of March was the best, with a fitting accuracy (coefficient of determination R^2^) as high as 0.79 and the smallest root mean square error (RMSE) of 96.17 μS cm^-1^; The fit was relatively worst in July, with a fitting accuracy R^2^ of only 0.58 and a root mean square error (RMSE) of 53.74 μS cm^-1^. The fit is also better in October, and the fitting accuracy R^2^ also reaches 0.60, while its root mean square error (RMSE) is also lower at 105.58 μS cm^-1^. The fit between the corrected best predictions and the measured values shows some variability across time, which is due to a number of reasons. Measurement errors caused by objective reasons such as ambient temperature, soil moisture and instrument sensitivity all have a certain impact on the experimental results and predicted values, which in turn cause some variability in the measured data and predicted results in different periods.

**Figure 8:**
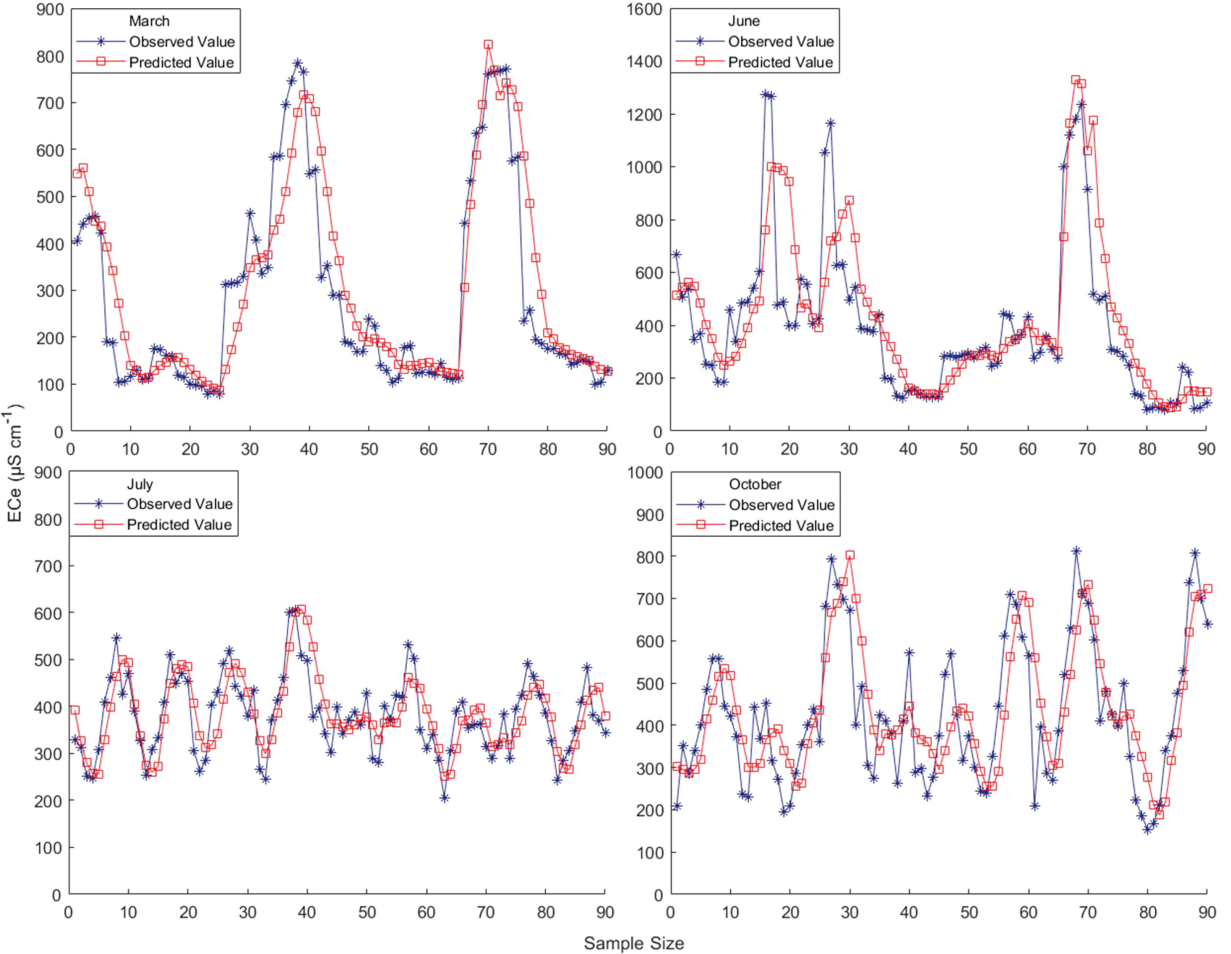
Measured and predicted values by period.

For the implementation of the Kalman filtering algorithm, the observation equations are used as the basis for further determining the state function, setting up the number of simulations (there are a total of 90 points in each period, so it is set to be 90, and the period is 5), the simulation time, the system state noise, and the observation noise, as well as further initializing the state equations, the output equations, and the covariance matrix. The initial value of the Kalman filter state is given and the error value is initialized and set to 0. On this basis, the operation can be carried out according to the idea of Kalman filter algorithm. In the operation process, the prediction of the state is needed first, and then the prediction error autocorrelation matrix is derived; Next, the Kalman gain solution is performed; Finally, state estimation and observation prediction can be obtained. After several iterative MATLAB operations, the corrected best predicted values were obtained. The simulated comparison between the measured values and the corrected best predicted values for different periods of time, and the correlation control plot between the two (see **Figure 8**).

As can be seen in **Figure 8**, overall, the fit between the corrected best predicted and measured values for different periods is better, with a high degree of approximation between the two. The Kalman filter algorithm was used to calibrate the multivariate linear model of measured conductivity and soil depth for each period, to find out the correlation between the measured and predicted values of the model for each period after calibration, and to compare the accuracy of the model with the accuracy of the model before calibration. The results of the comparison of the fitting accuracy between the corrected best prediction and the measured values are obtained for different periods.

**Figure 9:**
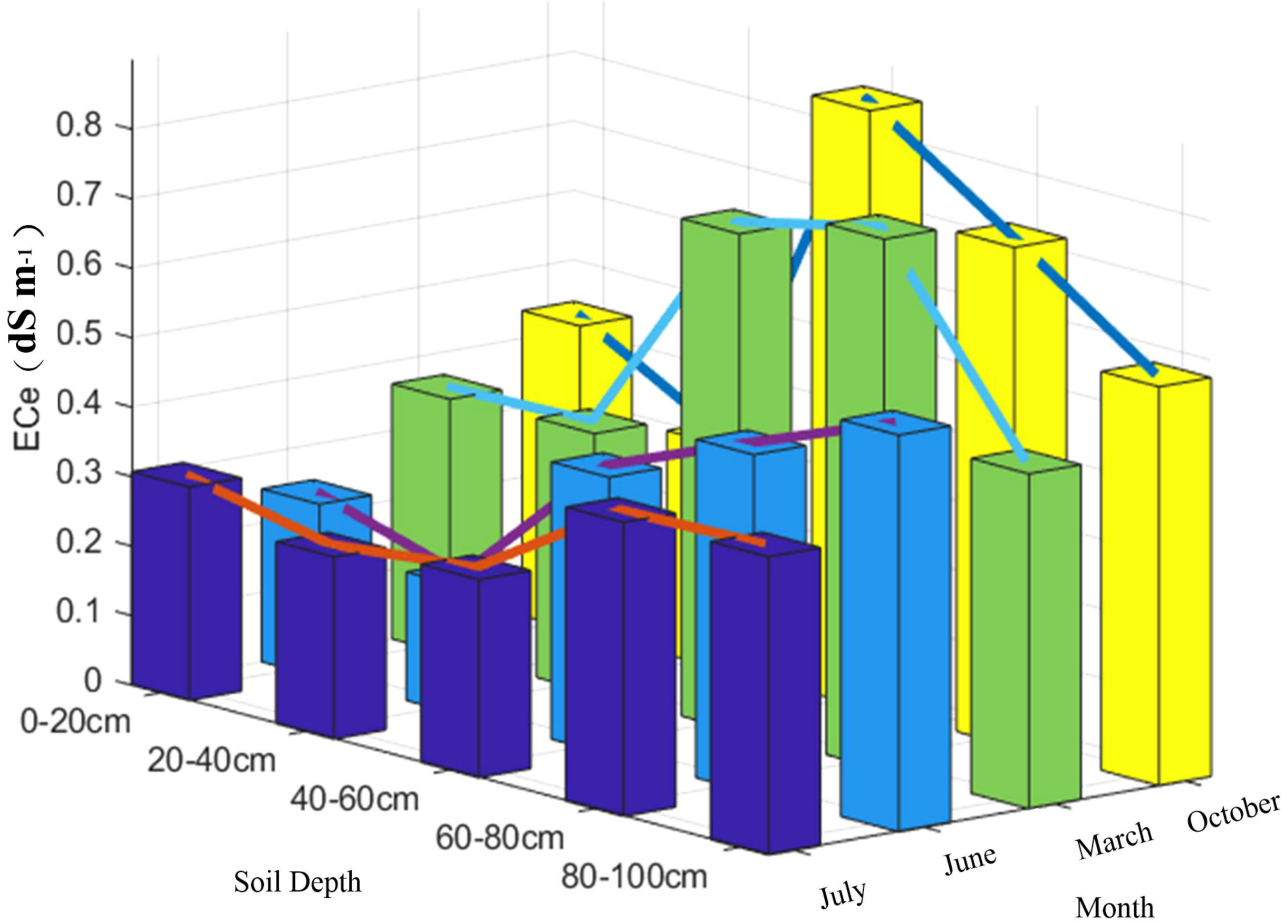
Salt accumulation process in soil layers in different months.

In order to further investigate the relationship between soil profile salinity and soil depth, the results of this research were combined with the results of previous studies. The predicted values obtained in each period were averaged according to the depth of the soil layer. In order to facilitate the comparison of the results, the overall effect of soil salinity accumulation between different months corresponded to July, June, March and October in descending order, and the related soil salinity accumulation process was plotted (see **Figure 9**).

As can be seen in **Figure 9**, soil conductivity showed a gradual accumulation trend at different periods. From the month of March, we can see that salts appeared to accumulate significantly in the soil layer from 40-80 cm and reached a peak value of 0.76 dS m^-1^ at 60-80 cm. Whereas, soil conductivity in October is peaked at 40-60 cm with a value of 0.86 dS m^-1^. However, from 40-80 cm, it is still a highly aggregated zone of salts at soil depth. In June and July salinity showed an aggregation with increasing soil depth, and this trend remained significant from 40-100 cm. Combined with the actual growth cycle of cotton and planting methods in South Xinjiang, the months of March, June, July and October can be defined as: pre-sowing, bud stage, boll stage and harvesting period, respectively. The months of June and July are the growing period of cotton, during which cotton is grown using drip irrigation, and it can be seen that the conductivity at 0-60 cm is significantly lower than in the other two months. However, as can be seen in **Figure 4**, soil salinity appeared to accumulate significantly in the 60-100 cm soil layer, both in June and July (growing period) and in March and October (non-growing period). Further explanation, although drip irrigation can wash the salt in all directions, but in the vertical direction, with the deepening of the soil layer, the washing effect will gradually weaken, and the salt will eventually accumulate in the deep soil. After the cotton harvest, it is necessary to carry out pre-winter deep ploughing, irrigation and alkali pressure, drainage and irrigation support, in order to achieve the purpose of salt washing and desalination. From **Figure 4**, it can be seen that before sowing in March, the conductivity from 0-40 cm was lower and less variable than that in October (harvesting period), with a mean value of 0.47 dS m^-1^. This further indicates that, after winter irrigation, the salinity of the soil surface was uniformly distributed, which is basically in line with the findings of Feng J et al. (Feng et al., 2021).

From the above research results, it can be seen that using the Kalman filter algorithm, the multivariate linear model can be corrected in order to improve the model prediction accuracy. Although the corrected model accuracy using Kalman filter algorithm facilitates the search and discovery of intrinsic patterns between soil profile salinity and soil depth. However, the initial model and its accuracy before correction are still important when using soil profile conductivity to study the real relationship between soil profile salinity and soil depth. This is because in Kalman filtering, the initial model and its accuracy determine the filter’s initial performance, convergence speed, and the benchmark for subsequent corrections. If the initial state estimate deviates too much from the true value, the filter will need more time or iteration steps to converge to the correct state, and it may also directly affect the filter’s performance and cause the system to diverge. Therefore, reasonably setting the initial state, error covariance, and model parameters can not only ensure the stability of the filter, but also provide a reliable basis for subsequent correction and optimization.

### 3.4 Application of data assimilation models in cotton yield and economic efficiency forecasting

**Figure 10:**
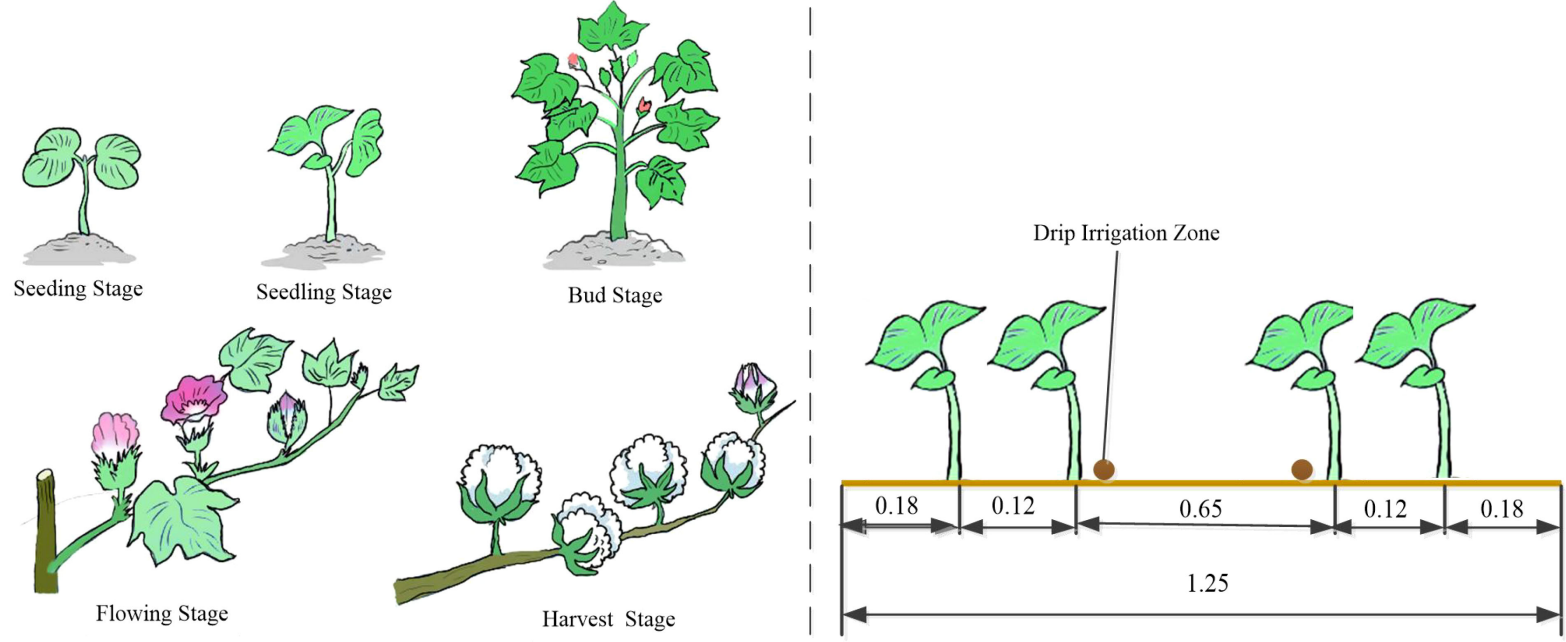
Cotton fertility period and drip irrigation belt arrangement (m).

The whole life span of cotton is the total number of days from the cotton seeding to the end of the flower harvest, about 210 days. It can be divided into five reproductive stages: seedling emergence, seedling stage, bud stage, boll stage and harvest stage (Lu et al., 2022). And Xinjiang because of the special geographic location, the use of under-membrane drip irrigation for planting, the study area has been used 1 membrane 2 tubes 4 rows of under-membrane drip irrigation technology planting cotton, in which the membrane width of 1.25 m, wide rows, narrow rows, as well as the distance between the membrane were 0.65 m, 0.12 cm and 0.2 cm, respectively, the drip irrigation belt laying (see **Figure 10**).

**Figure 11:**
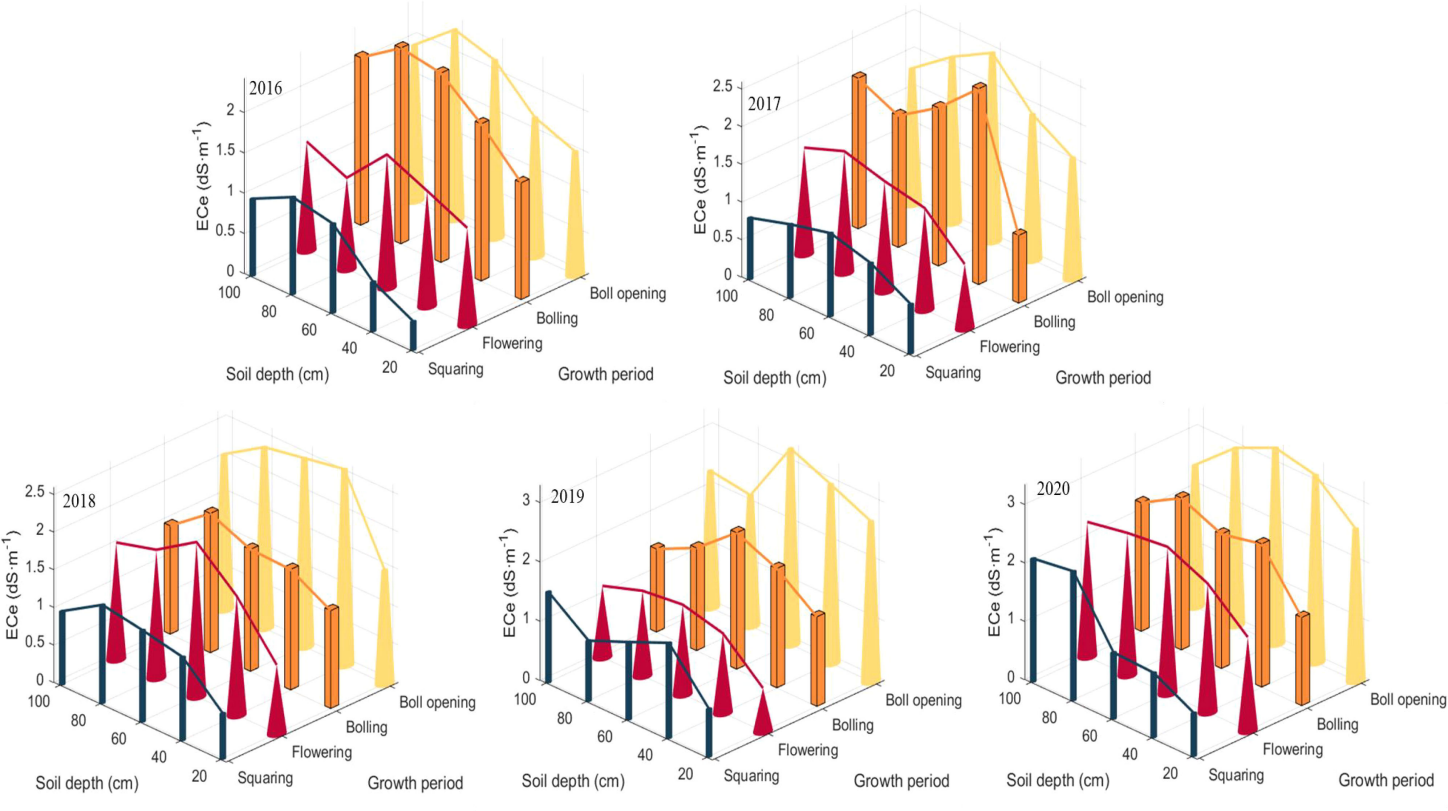
Salt accumulation processes in the 0-100 cm soil layer, 2016-2020.

Cotton goes through four key periods from nutrient growth to reproductive growth: seedling stage (April), bud stage (June), boll stage (July), and fluffing stage (August). Considering the sampling date, the information collected in the previous five years was utilized, so the bud stage and boll stage were selected to determine the best yield estimation factor for cotton (see **Figure 11**). The relevant information of Cotton Plant Height, Chlorophyll and Production are shown in **Table 3**.

**Table 3:** 2016-2020 cotton plant height, chlorophyll and seed cotton production.

Year	Plant height/cm	Chlorophyll/SPAD	Cotton seed yield/(kg·hm^-2^)
2016	84.65 ± 9.03	36.49 ± 1.74	4 984.37 ± 112.95
2017	83.31 ± 6.63	40.39 ± 4.35	5 355.61 ± 98.13
2018	82.63 ± 2.5	42.29 ± 3.41	5 229.44 ± 179.02
2019	67.31 ± 5.01	32.85 ± 2.0	4344.70 ± 115.10
2020	59.65 ± 2.51	31.78 ± 2.41	4046.56 ± 243.63

SPAD stands for Soil Plant Analysis Development.

In this study, the seed cotton yield of the first three years was used as the modeling set and the data of the last two years as the validation set, respectively. Different soil conductivity data at bud stage (June) and boll stage (July) were used as modeling factors to establish multivariate linear models.

**Table 4** shows the feasibility of multivariate linear modeling using different soil conductivities in June and July to predict seed cotton yield. Bringing the conductivity data predicted using data assimilation into the model yields 5203-5551 kg hm^-2^, while the study area is 15 hm^-2^, so the total production should be: 78048-83269 kg. According to the survey statistics of cotton purchase price of 2020 face of each regiment of Aral Reclamation Area, the purchase price of cotton is in the range of 4.88 - 5.34 RMB kg^-1^, the government subsidized price of 2.26 RMB kg^-1^, but the more frequent cotton purchase activities in the early and middle stage, the final farmers per kilogram of cotton revenue average price of about 7.15 RMB. And seeds, fertilizers, pesticides, mulch, drip irrigation belt, water, electricity, machine power, machine picking fee and other planting costs of about 32,250 RMB hm^-2^. Based on the statistical results of cotton unit price and planting costs in Alar Reclamation Area, combined with the cotton production in the study area, the study area in 2021, the revenue should be: 74,296.67-111,621.54 RMB.

**Table 4:** Cotton yield prediction model based on different critical periods.

Month	Model	R^2^	RMSE (kg·hm^-2^)
June	. $y=5630.70-157.7125x_{3}-33.93x_{4}$ .	0.98	1023.23
July	$y=5280.58+259.50x_{2}-338.78x_{5}$	0.99	1086.03

$x_{2}$ represents 20-40 cm conductivity data; $x_{3}$ represents 40-60 cm conductivity data; $x_{4}$ represents 60-80 cm conductivity data; $x_{5}$ represents 80-100 cm conductivity data; y represents cotton production.

## 4 Discussion and conclusions

### 4.1 Effectiveness of modeling factors

This study evaluated the suitability of different apparent conductivity modeling factors across various soil depths, using the EM38-MK2 geophysical conductivity meter. By comparing the performance of different modeling factors in multiple measurement modes, the most effective factors were identified for each depth range. The findings of the present study are in agreement with those of some previous studies using conductivity instruments for soil salinity prediction, while also presenting new insights. Although, Song and Zhao et al. (Zhao et al., 2013; Song et al., 2019) have used EM38 and combined with soil measured conductivity data (ECe) to analyze salinization at different soil depths and achieved better research results. However, it is modeled by selecting the apparent conductivity data (ECa) at a specific soil depth, at the same depth, and in different measurement modes (horizontal and vertical modes), in order to determine which mode has applicability for ECa. Instead, this paper is not limited to the measurement model, but is modeling ECe at different soil depths using ECa at different depths and in different modes, respectively, to determine the most effective factor in each depth range. Compared to Song and Zhao et al, this study does not need to re-collect ECa at a specific soil depth, and extends the scope of consideration, which has the advantage of rapidity and simplicity, and saves financial and material resources. As for the research results, Sun et al. (Sun et al., 2012) had used EM38 to identify heavily saline soils and found that EM38 horizontal mode measurements were more sensitive to shallow soils, while vertical mode measurements were more sensitive to deep soils, which was further verified by the results of the present study, especially in the depth ranges of 0-40 cm and 80-100 cm, with EC_h0.5_ in the horizontal mode and EC_v1.0_ in the vertical mode The performance was superior and the results of this study were reliable. The reasons for the consistency are as follows: First, the research areas of this article and the scholars are both in the Tarim Basin in southern Xinjiang, where rainfall is scarce, evaporation is strong, and soil salinization is serious. Secondly, EM38 is used to predict soil salinity in different soil layers with the help of apparent conductivity. The selected methods are all linear modeling, and the fitting effect is significant.

In this paper, by optimizing the sampling strategy based on the performance of different modeling factors, the accuracy of the soil salinity prediction model has been significantly improved, reducing the need for labor-intensive field sampling, but there are some limitations. The data were mainly derived from samples under a single climatic condition, so the broad applicability of the model may be limited. The results of this study have yet to be validated under a wider range of regional and climatic conditions than those of Mello et al. (Mello et al., 2022) who used datasets with different lithologies, landforms and soil types. Future research should focus on improving the accuracy of global models for large-scale applications, potentially through the integration of other methods, such as multiple regression and covariance modeling, to enhance prediction accuracy and extend these findings to broader geographic regions.

### 4.2 The advantages of the Kalman filter algorithm

The Kalman filtering algorithm was called LLMS (Linear Least Mean Squares) when it was first proposed, because it optimizes a linear stochastic system with noise-containing sensor measurements in a least-square manner to obtain an optimal solution. In other words, it optimizes the collected data that do not contribute or interfere with the system. As for the application of Kalman filter algorithm, some scholars have long applied it to soil moisture forecasting and achieved good research results (Gruber et al., 2019). The vast majority of scholars use Kalman filter algorithm research object in soil moisture, for with the help of soil apparent conductivity data, the study of soil salinization results is less. Huang et al. (Huang et al., 2017a) have used electromagnetic conductivity imaging and ensemble Kalman filter to monitor and model soil water dynamics. The results of EnFK modeling (Lin’s concordance = 0.89) were superior to the physical model, and superior or equivalent to the empirical model (Artificial Neural Networks) on loamy, clayey, and biphasic soil profiles. This shows the feasibility of combining soil apparent conductivity data with the ensemble Kalman algorithm. While the study area of this paper is arid saline soil and the soil salinity prediction model is a linear model, the Kalman filter algorithm is preferred to calibrate and validate the model to verify the feasibility of the scheme. The results showed that the Kalman filter method significantly improved the accuracy of the model. Although the corrected R²values for June and October were slightly higher than those for July (0.59 and 0.60, respectively), the model showed the greatest improvement in July, with an 81.3% increase in R². However, the intense heat and high temperatures in July may have interfered with the sensitivity of the instrument, resulting in the worst model performance during this period, with an R² of only 0.58 and a maximum RMSE of 337.22 μS cm^-1^. The model performance for the month of March was also higher than that for the month of October (0.59 and 0.60, respectively). In March, on the other hand, its coefficient of determination (R²) reached 0.79, which is higher than the values of the other months, but the R²of its linear model is 0.78, and the linear model calibrated by the Kalman filter algorithm is equivalent to the linear model. It can be seen that compared with the traditional model, the Kalman filter algorithm is able to dynamically adjust the model predictions so as to continuously improve the predicted values according to the observed data.

Although, the results of these studies show that the Kalman filter method algorithm can be an important tool to improve the accuracy of soil salinity prediction, especially when applied to the linear relationship between soil depth and salinity content. However, there are still some limitations of the research results. First, the study area of this method is extremely arid salinized soil. The feasibility of this idea needs to be further verified if the salinization degree of the soil is studied in a non-arid area. Secondly, the prerequisite salt prediction model using Kalman filter algorithm is a linear model, and once the model is nonlinear, the algorithm has to be re-selected for validation (Huang et al., 2017b). Therefore, future research will have to explore the selection of suitable data assimilation algorithms according to the actual situation.

### 4.3 Implications for cotton cultivation

Cotton production areas in South Xinjiang are deeply affected by soil salinization, which on the one hand will lead to soil loss of fertility, crop yield reduction; on the other hand, it will also have a great impact on the ecological environment (Li et al., 2014). Therefore, the monitoring, management and improvement of salinized farmland is particularly important, and how to obtain soil salinity information quickly, accurately and in real time is the basic premise of management and improvement of farmland. In this paper, Kalman filter algorithm is applied to the multivariate linear model of conductivity and soil depth, which greatly improves the accuracy of the model. The relationship between soil profile salinity and depth was clearly established, and the model predictions were validated by comparing the measured and predicted conductivity values. For the spatial and temporal scale changes of salinity in the 0-100 cm soil layer of drip-irrigated cotton under the membrane at different times, the salinity in the 0-20 cm soil layer in the vertical direction at the inter-membrane site was less, and the salinity in the 60-100 cm layer was accumulated to a greater extent. Drip irrigation water washed the salts in all directions, and in the vertical direction, as the soil layer deepened, the washing effect gradually weakened, and the salts eventually accumulated in the deep soil layer. Although drip irrigation water has a significant leaching effect on salts, but this is a certain number of times and short-lived (Zhao et al., 2016). In contrast, under strong transpiration, the root system of cotton absorbs water continuously, and the root system absorbs water so that salts are transported to the root system. Although cotton can absorb and utilize some of the trace elements in brackish water, most of the salts remain in the root zone.

The numerical simulation results of this study showed that the 80-100 cm soil layer was relatively stable with a small range of salinity dynamics due to less water infiltration. In the 40-60 cm soil layer, the salinity is significantly higher than that in the 0-20 cm layer, and the deep soil water and salt have a tendency to migrate to the bare surface at the edge of the membrane due to evaporation and root absorption. In this study, we found that soil salts accumulated during the reproductive period of cotton fluctuated in a sawtooth pattern, and showed a gradual accumulation trend. This is due to the increase in soil water content after irrigation led to a transient decrease in the salinity of each soil layer, and then soil water gradually evaporated and dissipated as well as being absorbed and utilized by cotton, resulting in a gradual rebound of soil salinity (Liu et al., 2013); Drip irrigation is effective in reducing salinity in the upper soil layer (0-60 cm), but salts tend to accumulate at greater depths over time, suggesting the need for additional salt management practices such as deep plowing, winter irrigation, and drainage. Generally, no irrigation is required during the seedling stage, but when the relative soil moisture content is lower than 50%, small irrigation will be carried out; when the relative soil moisture content is lower than 60% during the bud stage, timely irrigation and topdressing of water-soluble fertilizers are required. Generally, irrigation is carried out 1-2 times during this period, and the irrigation cycle is 10-12 days; when the relative soil moisture content is lower than 70% during the flower and boll stage, timely irrigation and topdressing of flower and boll fertilizers are required. Generally, irrigation is carried out 8-10 times during this period, and the irrigation cycle is 7-10 days. After the full boll stage, the amount of irrigation gradually decreases, and irrigation will be stopped before defoliation. However, during the growth period, specific measures must be taken according to the specific situation. For example: if the temperature is high in autumn, the water stop time must be appropriately postponed (Datta et al., 2019). These measures can help prevent salt accumulation at deeper depths and support the sustainable cultivation of cotton in arid regions. And this study can better simulate the spatial and temporal distribution of soil salinity in drip-irrigated cotton fields and predict the yield and production of cotton fields, but in the future simulation process need to take into account the factors of soil evaporation, plant transpiration and root water uptake, so that the more reasonable optimization of the model parameters, the closer the simulation results are to the actual measurement values (Li et al., 2019).

## Data Availability

The datasets presented in this article are not readily available because Our data has taken a lot of time and effort, and there is still research going on, and the data is confidential. Requests to access the datasets should be directed to qingsongjiang219@126.com.
